# Vascular uptake on ^18^F-FDG PET/CT predicts relapse in new-onset PMR

**DOI:** 10.1093/rheumatology/keaf557

**Published:** 2025-10-23

**Authors:** Ningqi Dai, Xinyi Sun, Linzi Meng, Jialin Teng, Chengde Yang, Xiaoyue Chen, Huihui Chi

**Affiliations:** Department of Rheumatology and Immunology, Ruijin Hospital, Shanghai Jiao Tong University School of Medicine, Shanghai, China; Department of Nuclear Medicine, Ruijin Hospital, Shanghai Jiao Tong University School of Medicine, Shanghai, China; Department of Rheumatology and Immunology, Ruijin Hospital, Shanghai Jiao Tong University School of Medicine, Shanghai, China; Department of Rheumatology and Immunology, Ruijin Hospital, Shanghai Jiao Tong University School of Medicine, Shanghai, China; Department of Rheumatology and Immunology, Ruijin Hospital, Shanghai Jiao Tong University School of Medicine, Shanghai, China; Department of Nuclear Medicine, Ruijin Hospital, Shanghai Jiao Tong University School of Medicine, Shanghai, China; Department of Rheumatology and Immunology, Ruijin Hospital, Shanghai Jiao Tong University School of Medicine, Shanghai, China

**Keywords:** PMR, GCA, ^18^F-FDG PET/CT, relapse

## Abstract

**Objective:**

In this study, we utilize ^18^F-fluorodeoxyglucose (FDG) PET/CT to assess vascular involvement in patients with PMR, aiming to clarify its role in identifying subclinical GCA and its potential association with disease relapse.

**Methods:**

We conducted a retrospective cohort study of 80 patients diagnosed with PMR who underwent PET/CT scanning at Ruijin hospital from September 2015 to December 2023. Based on PET/CT findings, patients were divided into two groups: those with vascular involvement and those without. We compared baseline clinical characteristics, treatments and relapse rates between the two groups. Multivariate Cox regression analysis was performed to identify independent predictors of relapse.

**Results:**

In our cohort, 14 patients (14/80, 17.5%) were identified as having subclinical GCA, with 93% (13/14) exhibiting extracranial vessel involvement. Relapse rates were significantly higher in patients with subclinical GCA compared with those with isolated PMR (71% vs 29%, *P* = 0.003). Vascular involvement was identified as a strong predictor of relapse with a hazard ratio (HR) of 3.731 (95% CI: 1.705–8.164). Conversely, arthralgia appeared to be a protective factor against relapse with a HR of 0.325 (95% CI: 0.148–0.711).

**Conclusion:**

Our findings highlight vascular involvement as a critical predictor of relapse in PMR, underscoring the utility of ^18^F-FDG PET/CT in detecting subclinical GCA and predicting prognosis. Further research is needed to establish screening protocols and optimize relapse prevention strategies in this population.

Rheumatology key messages

- PMR patients with subclinical GCA exhibited a high prevalence of extracranial vascular involvement and significantly increased relapse rates.
- ^18^F-FDG PET/CT is a valuable tool for assessing vascular involvement and predicting the risk of relapse in patients with PMR.

## Introduction

PMR is a common rheumatic disease in the elderly, characterized by disabling pain and stiffness predominantly in the shoulders and hips, and pathologically marked by musculotendinous and periarticular connective tissue inflammation that may also involve the spine and peripheral joints [1, 2]. Additionally, 40–50% of patients may experience constitutional symptoms, including fever, fatigue, weight loss and limited joint mobility [3]. While oral glucocorticoid therapy is effective in alleviating symptoms [4], tapering or discontinuing the medication is challenging for some patients, with recurrence rates ranging from 27.6% to 55.0% [5–8].

PMR and GCA have long been considered as two different but frequently overlapping conditions. About 40–60% of patients diagnosed with GCA have PMR, while 10–16% of PMR patients may present with concomitant GCA [9–11]. Recent studies on subclinical GCA have provided deeper insights into the relationship between GCA and PMR. They emphasize the challenges in defining the boundaries of these diseases and have led some researchers to propose that GCA and PMR may exist along a continuum, manifesting independently, concurrently or sequentially [12].

In clinical practice, GCA is an urgent and severe condition, accompanied with a high risk of permanent visual loss and other ischaemic complication, which require higher doses of glucocorticoids and more aggressive immunosuppressive therapies [13, 14]. In contrast, PMR typically presents with less severe symptoms and a relatively more favourable prognosis. However, the overlapping situation, e.g. PMR patients with subclinical GCA—defined as vascular inflammation detected by imaging or histology in the absence of overt vasculitic symptoms—remains under-recognized, and optimal management strategies for this group are yet to be established [15]. In 2001, Marzo-Ortega *et al.* reported a case of PMR with subclinical vasculitis who was difficult to treat and first discussed the close link between underlying vasculitic process and prognosis in PMR [16]. Subsequently, a growing body of research has focused on this clinical phenomenon. Imaging modalities, such as US, ^18^F-fluorodeoxyglucose (FDG) PET/CT and magnetic resonance angiography (MRA), have shown promise in detecting vascular inflammation [17]. However, the necessity of routine vascular screening in PMR patients, the most appropriate screening methods and the optimal treatment strategies for PMR patients without concurrent GCA symptoms remain controversial in current clinical practice.

Given this background, we conducted a retrospective analysis of PMR patients at our institution over the past decade, including those who underwent PET/CT as part of their initial diagnostic evaluation, primarily to assess for large-vessel vasculitis and exclude underlying malignancy. This study aims to compare the baseline clinical characteristics and prognostic outcomes of patients with and without subclinical GCA. We seek to better understand the patterns of vascular involvement in PMR patients with subclinical GCA, assess its prognostic implications and explore the potential need for routine vascular screening in these patients.

## Methods

### Study population

We conducted a retrospective analysis of patients diagnosed with PMR who underwent ^18^F-FDG PET/CT at Ruijin Hospital, Shanghai Jiao Tong University School of Medicine, between September 2015 and December 2023. Clinicians identified patients with PMR based on clinical manifestation and laboratory findings, and PET/CT was recommended for suspected cases to evaluate for large-vessel vasculitis and to rule out malignancy when feasible. All included patients met the 2012 American College of Rheumatology (ACR)/European Alliance of Associations for Rheumatology (EULAR) classification criteria for PMR [18]. Only patients who had received a limited course of glucocorticoid treatment <20 mg/day of prednisone or its equivalent (<14 days and ≤20 mg/day of prednisone or its equivalent) and no history of immunosuppressive therapy prior to systematic evaluation were included. Furthermore, to ensure consistency in clinical outcomes, only those with regular follow-up visits for at least the first 12 months of treatment were considered for inclusion. Patients with a history of prior rheumatic diseases, malignant tumours or symptoms suggestive of GCA (headache, jaw claudication, sudden-onset visual disturbances, limb claudication, etc.) were excluded. Furthermore, patients unable to provide information on relapse were also excluded.

At baseline, comprehensive clinical, laboratory and imaging data, including the results of ^18^F-FDG PET/CT scans, were collected to assess vascular involvement. Follow-up data were also retrospectively collected for relapse rates and disease progression. Relapse was defined as the recurrence of PMR symptoms with at least one elevated inflammatory marker (e.g. CRP or ESR), and further required either an increase in the glucocorticoid dose and/or immunosuppressive agents. All relapse events were confirmed by an experienced rheumatologists following the exclusion of alternative diagnoses, such as infection or malignancy. This study was approved by the Institutional Review Board of Ruijin Hospital (ID: 2024–401) and was conducted in accordance with the principles of the Declaration of Helsinki. Informed consent was obtained from all participants.

### ^18^F-FDG PET/CT examination

All patients fasted for at least 6 h prior to the administration of ^18^F-FDG. PET/CT was performed 50–90 min after intravenous injection of ^18^F-FDG at a dose of 0.10–0.12 mCi/kg. Two devices were used for PET/CT scan: the Discovery MI (GE Healthcare), with a scanning area range from the top of the head to the mid-calf; and the uEXPLORER (United Imaging Healthcare), with a scanning ranged from the top of the head to feet. Image reconstruction was performed using the ordered subset expectation maximization (OSEM) algorithm, with all standard corrections applied.

Two experienced nuclear medicine physicians independently reviewed the ^18^F-FDG PET/CT images of all PMR patients. In cases of disagreement between the two nuclear medicine physicians, a third experienced physician would be consulted for final adjudication. They assessed FDG uptake in the superficial temporal arteries and extracranial vessels, including the ascending aorta, aortic arch, thoracic aorta, abdominal aorta, subclavian arteries, axillary arteries, common carotid arteries, common iliac arteries and femoral arteries. The maximum standardized uptake value (SUVmax) at these sites was recorded. Vascular involvement was visually evaluated using a previously reported grading system (0–3): grade 0 (≤mediastinum, no FDG uptake); grade 1 (<liver, minimal but not negligible FDG uptake); grade 2 (=liver, clearly increased FDG uptake) and grade 3 (>liver, markedly increased FDG uptake) [19]. Patients who exhibited FDG uptake graded 2 or higher in any vascular segment were classified as having vascular involvement and were further categorized as having subclinical GCA.

In addition, visually assessed FDG uptake in 12 musculoskeletal regions were also assessed, including the cervical and lumbar spinous processes, left and right sternoclavicular joints, shoulders, hips, ischial tuberosities and greater trochanters. Each region was scored using a standardized three-point scoring system: 0 (no FDG uptake), 1 (moderate uptake, less than liver) or 2 (intense FDG uptake, equal to or greater than liver). The total Leuven score was calculated by summing the scores across all 12 regions.

### Statistical analysis

A descriptive analysis of the data was performed. Continuous variables were expressed as the mean ± S.D. for normally distributed data and as the median (interquartile range, IQR) for non-normally distributed data, with comparisons using Student’s *t*-test or Mann–Whitney *U*-test, depending on normality. Categorical variables were presented as frequencies and percentages and compared using *χ*^2^ test or Fisher’s exact test, depending on sample size and expected frequencies. All analyses were performed using complete data without imputation, as the proportion of missing data was minimal and did not affect the robustness of the findings. To identify predictors of relapse, Cox proportional hazards regression was employed. A variance inflation factor (VIF) test was performed to detect potential multicollinearity among independent variables, with a threshold of VIF > 5 considered indicative of significant multicollinearity. Univariate Cox regression was conducted for demographic and clinical variables, and those with a *P*-value ≤0.10 were considered for inclusion in the multivariate model using a backward elimination method. The cumulative probability of relapse was analysed using Kaplan–Meier curves, and differences between the groups were assessed with the log-rank Mantel–Cox test. Statistical significance was defined as two-tailed *P* < 0.05. All statistical analyses were performed using IBM SPSS Statistics, version 26.0 (IBM Corp, Armonk, NY, USA).

## Results

**Figure 1.**
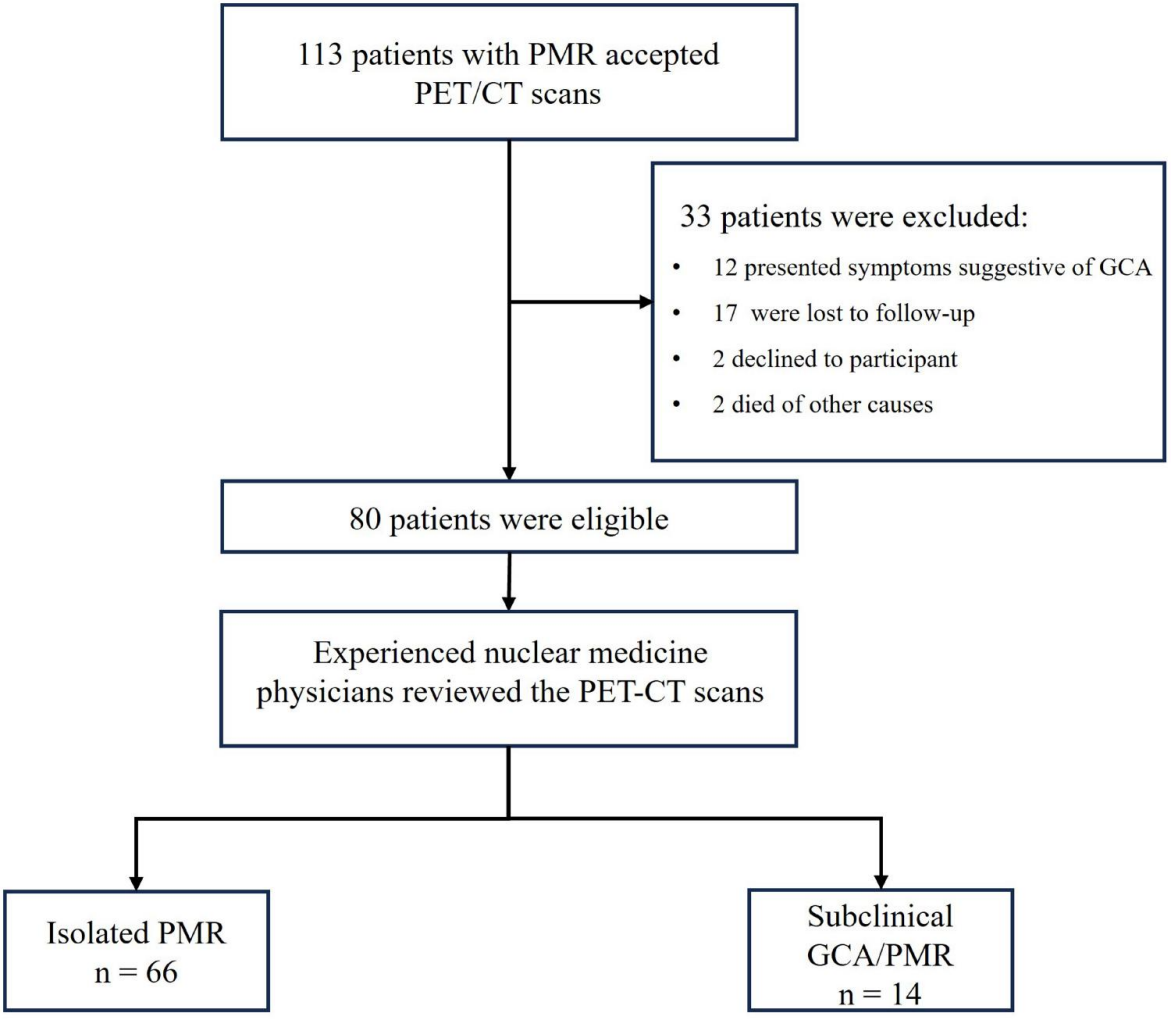
Flowchart of the enrolment process

A total of 80 PMR patients were included in the study and divided into two groups based on arterial FDG uptake: the isolated PMR group (*n* = 66) and the subclinical GCA/PMR group (*n* = 14) (Fig. 1). The maximum FDG uptake grade across vascular segments for each patient is presented in Supplementary Table S1. Baseline demographic features, including age and sex, were comparable between the two groups. Subclinical GCA patients had a higher prevalence of constitutional symptoms, particularly fatigue (43% vs 12%, *P* = 0.013). The difference in musculoskeletal involvement, CRP and ESR were minimal between the groups. Table 1 summarizes the clinical characteristics of the included patients. Visual assessments of the 12 musculoskeletal regions revealed no significant differences in FDG uptake between the subclinical GCA/PMR group and the isolated PMR group, except for the left hips. Overall, no statistically significant differences were observed in total Leuven scores between the two groups as shown in Supplementary Fig. S1.

**Table 1.** Clinical characteristics in isolated PMR and PMR with subclinical GCA

	Isolated PMR (*n* = 66)	Subclinical GCA/PMR (*N* = 14)	*P*-value
Age, years, mean ± S.D.	69.5 ± 8.4	66.0 ± 9.4	0.180
Female sex, *n* (%)	53 (80)	12 (86)	1.000
CRP, mg/l, median (IQR)	31.5 (55.2)	38.8 (67.1)	0.795
ESR, mm/h, median (IQR)	72.5 (35.5)	56.0 (39.5)	0.425
Constitutional symptoms			
Fever, *n* (%)	24 (36)	8 (57)	0.149
Fatigue, *n* (%)	8 (12)	6 (43)	0.013
Anorexia, *n* (%)	2 (3)	2 (14)	0.139
Weight loss, *n* (%)	10 (15)	2 (14)	1.000
Musculoskeletal involvement			
Arthralgia, *n* (%)	45 (68)	8 (57)	0.428
Myalgia, *n* (%)	48 (73)	10 (71)	0.921
Limited range of joint motion, *n* (%)	53 (80)	12 (86)	0.638
Morning stiffness, *n* (%)	39 (59)	7 (50)	0.532
Treatment			
GC dose, mg, mean ± S.D. (equivalent to prednisone)			
Baseline	15.34 ± 7.01	17.5 ± 6.43	0.292
Month 6	6.55 ± 3.58	10.89 ± 8.8	0.003
Months 12	3.75 ± 2.88	7.14 ± 4.26	0.012
Time to low-dose GC (≤5 mg prednisone per day), months, median (IQR)	6 (8.25)	10.5 (18)	0.005
Duration of GC, months, median (IQR)	26 (33.25)	30 (35)	0.954
Methotrexate, *n* (%)	50 (76)	13 (92)	0.143
Leflunomide, *n* (%)	7 (11)	1 (7)	1.000
Tofacitinib, *n* (%)	19 (29)	2 (14)	0.334
Relapse, *n* (%)	19 (29)	10 (71)	0.003

GC, glucocorticoid; *n*, number of cases; IQR, interquartile range.

Baseline glucocorticoid dose, duration of therapy and use of DMARDs were comparable between the two groups. However, patients in the subclinical GCA/PMR group received significantly higher glucocorticoid doses at both 6 months (10.89 ± 8.8 vs 6.55 ± 3.58, *P* = 0.005) and 12 months (7.14 ± 4.26 vs 3.75 ± 2.88, *P* = 0.012), indicating a slower tapering process. In addition, the recurrence rate was significantly higher in the subclinical GCA group compared with the isolated PMR group (71% vs 29%, *P* = 0.003). No severe vascular complications, such as aortic aneurysm formation or anterior ischaemic optic neuropathy (AION), were observed in the subclinical GCA group during follow-up.

**Figure 2.**
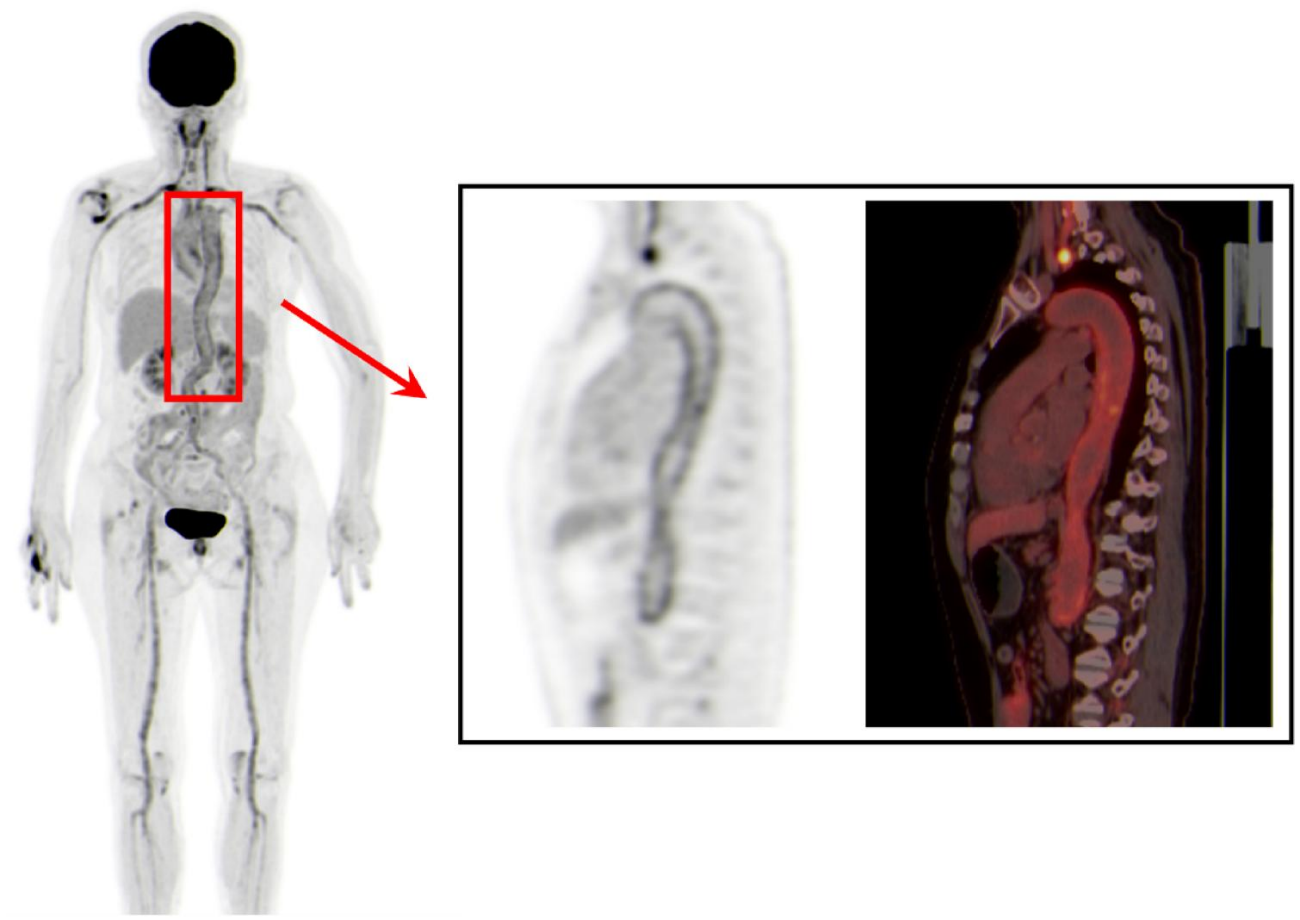
FDG-PET whole-body maximum intensity projection (MIP) image (left) and sagittal PET (right) show increased FDG uptake in the thoracic aorta

The distribution of vascular involvement in 14 patients with subclinical GCA evaluated using ^18^F-FDG PET/CT is described in Table 2. The most frequently involved regions were the thoracic aorta (93%) and abdominal aorta (86%), followed by the ascending aorta (86%) and aortic arch (71%). Extracranial arteries, including the common carotid (71%), subclavian (50%), axillary (43%) brachiocephalic trunk (43%), also exhibited significant FDG uptake. In contrast, temporal artery involvement was rare (7%), indicating a predominantly extracranial pattern. Fig. 2 illustrates increased FDG uptake in the thoracic aorta from the whole-body maximum intensity projection (MIP) of one patient. Interestingly, no cases of isolated cranial artery involvement were observed, with only one instance of mixed involvement, differing from findings from previous studies [20, 21].

**Table 2.** Distribution of vascular involvement in patients with PMR and subclinical GCA

Sites	(*n* = 14)
Aorta, *n* (%)	13 (93)
Ascending aorta, *n* (%)	12 (86)
Aortic arch, *n* (%)	10 (71)
Thoracic aorta, *n* (%)	13 (93)
Abdominal aorta, *n* (%)	12 (86)
Brachiocephalic trunk, *n* (%)	6 (43)
Common carotid, *n* (%)	10 (71)
Subclavian, *n* (%)	7 (50)
Axillary, *n* (%)	6 (43)
Temporal, *n* (%)	1 (7)
Common iliac, *n* (%)	5 (36)
Femoral, *n* (%)	2 (14)

**Figure 3.**
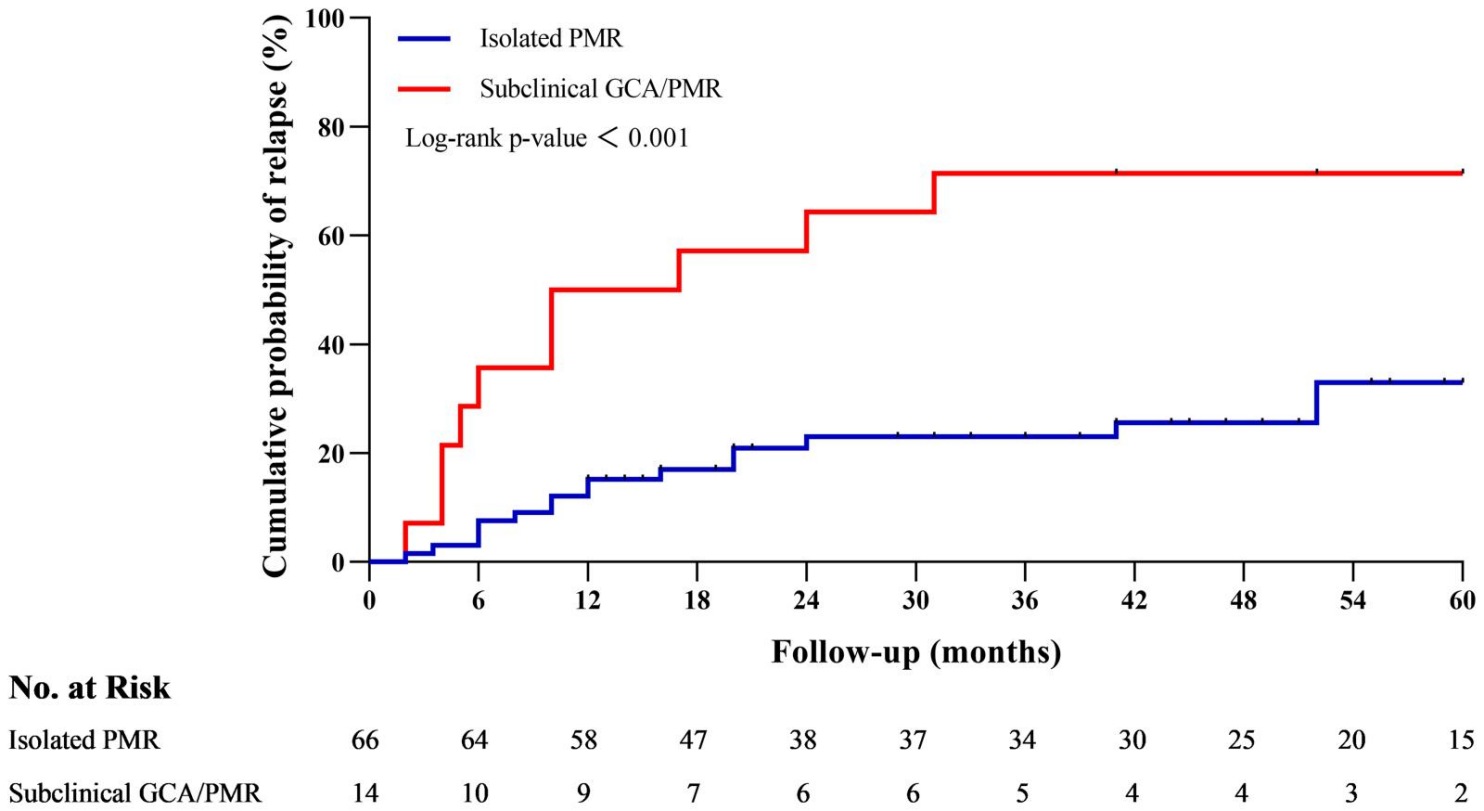
Kaplan–Meier survival curves of relapse in patients with isolated PMR and subclinical GCA/PMR, Log-rank *P*-value, and number at risk in the follow-up (months)

The Kaplan–Meier survival curve illustrates the cumulative probability of relapse in patients with isolated PMR vs those with subclinical GCA/PMR over a 60-month follow-up period (Fig. 3). Patients with subclinical GCA/PMR, showing a significantly higher relapse rate compared with the isolated PMR patients (log-rank *P*-value <0.001). The one-year relapse rates for the isolated PMR group and the subclinical GCA/PMR group were 15.4% (95% CI, 10.9–19.9%) and 50.0% (95% CI, 36.6–63.4%), respectively. The five-year relapse rates were 33.8% (95% CI, 19.2–48.4%) and 71.4% (95% CI, 47.7–95.1%), respectively. Cox proportional hazards regression analysis was performed to identify predictors of relapse (Table 3). In the univariate analysis, vascular involvement emerged as the strongest independent risk factor (HR 3.731, 95% CI, 1.705–8.164), alongside other potential predictors such as age (HR 0.961, 95% CI, 0.921–1.003), fatigue (HR 2.355, 95% CI, 1.030–5.386), limited range of joint motion (HR 6.890, 95% CI, 0.933–50.904) and were identified as potential predictors of relapse. Notably, arthralgia was identified as a protective factor (HR 0.433, 95% CI, 0.203–0.926), reducing the risk of relapse. In the multivariate Cox regression model, only vascular involvement (HR 3.070; 95% CI, 1.385–6.808), arthralgia (HR 0.325; 95% CI, 0.148–0.711) and limited range of joint motion (HR 7.163; 95% CI, 0.931, 55.110) remained significant predictors of relapse.

**Table 3.** Association of patient characteristics and relapse in univariable and multivariable analysis

Parameter	Univariate analysis				Multivariate analysis			
	*β*	*P*-value^a^	HR	95% CI	*β*	*P*-value	HR	95% CI
Age, years	−0.040	**0.066**	0.961	0.921, 1.003				
Sex, female	−0.253	0.641	0.776	0.268, 2.251				
CRP, mg/l	−0.003	0.444	0.997	0.988, 1.005				
ESR, mm/h	−0.005	0.452	0.995	0.982, 1.008				
Fever	0.483	0.210	1.620	0.761, 3.448				
Fatigue	0.857	**0.042**	2.355	1.030, 5.386				
Weight loss	0.018	0.974	1.018	0.351, 2.949				
Arthralgia	−0.836	**0.031**	0.433	0.203, 0.926	−1.124	0.005	0.325	0.148, 0.711
Myalgia	0.613	0.217	1.845	0.698, 4.881				
limited range of joint motion	1.930	**0.059**	6.890	0.933, 50.904	1.969	0.059	7.163	0.931, 55.110
Morning stiffness	0.551	0.154	1.735	0.814, 3.698				
Vascular involvement	1.317	**0.001**	3.731	1.705, 8.164	1.122	0.006	3.070	1.385, 6.808
Glucocorticoids dose baseline, mg/day	0.023	0.358	1.023	0.974, 1.074				

a The bold text indicates variables with *P*-values <0.10 in the univariate analysis and included in the multivariate analysis.

## Discussion

This study demonstrates the pivotal role of vascular involvement detected by ^18^F-FDG PET/CT in predicting relapse in PMR patients. Vascular involvement on PET/CT emerged as the strongest independent predictor of relapse, with patients classified as having GCA exhibiting significantly higher one-year and five-year relapse rates than isolated PMR. The pattern of vascular involvement was predominantly extracranial, particularly affecting the thoracic and abdominal aorta, underscoring the diagnostic value of PET/CT in identifying subclinical GCA and stratifying relapse risk and its utility for relapse risk stratification.

Importantly, this study also contributes novel data from an East Asian cohort, where PMR incidence is lower and imaging-based studies particularly on PET/CT remain limited [22, 23]. We report for the first time a 17.5% prevalence of subclinical GCA among Chinese PMR patients, which is slightly lower than the 29% reported in European PET/CT-based studies [24]. This discrepancy may be attributed to the difference in the study populations. While our study focused exclusively on PMR patients who met strict inclusion criteria, excluding those with symptoms suggestive of GCA, they may have included patients with overlapping clinical features or undiagnosed GCA. Moreover, variations in demographic characteristics, such as age and underlying comorbidities, may also have influenced the observed rates of vascular involvement.

The concept of subclinical GCA, defined by vascular involvement on imaging in the absence of classical ischaemic symptoms, remains challenging, particularly in the context of overlapping features between GCA and PMR [15]. Although we excluded patients presenting with symptoms suggestive of overt GCA, most patients with vascular involvement exhibited constitutional symptoms, particularly fever and fatigue. These symptoms are not specific and may also occur in isolated PMR. The prevalence of such systemic symptoms in subclinical GCA in our cohort was comparable to that reported in PMR patients from other of East Asian studies, suggesting that these manifestations may not reliably distinguish GCA from PMR in this population [25, 26]. Still, we acknowledge that the presence of systemic symptoms introduces some degree of diagnostic ambiguity, which should be considered when interpreting our findings.

PMR and GCA are increasingly recognized as conditions along a shared disease spectrum. This spectrum theory is supported by prior studies, which propose that subclinical GCA may represent an intermediate state, bridging isolated PMR and classical GCA. In our study, the overall relapse rate was 36.2%, consistent with previously reported rates in PMR patients [5–8]. We found patients with subclinical GCA demonstrated significantly higher relapse rates, reinforcing the need for vascular screening to stratify relapse risk and guide personalized treatment. Cox regression analysis identified vascular involvement as a significant risk factor for relapse (HR 3.731, 95% CI, 1.705–8.164), consistent with the findings of De Miguel *et al.* [27]. Another retrospective study of 337 patients by Moreel *et al.* reported that PMR patients with vascular involvement on PET/CT did not exhibit a higher relapse rate compared with those with isolated PMR [28]. Notably, in their cohort, patients with vascular involvement received higher glucocorticoid doses initial and during the first 12 months after diagnosis, whereas in our cohort, the glucocorticoid doses were comparable between the two groups, yet the subclinical GCA group showed a significantly higher relapse rate. This discrepancy in treatment intensity may account for the differing relapse rates between the two studies. Taken together, these findings suggest that patients with subclinical GCA may require higher glucocorticoid doses to reduce the risk of relapse. Prospective studies are warranted to further define optimal treatment regimen for this subgroup. What is more, these results highlight the importance of integrating vascular assessment into the clinical management of PMR, particularly for identifying high-risk patients who may benefit from more intensive monitoring and treatment.

There is currently no consensus on whether all patients with clinically isolated PMR should undergo imaging-based screening for subclinical GCA or the optimal method for evaluating vascular involvement. While US of the temporal and axillary arteries is recommended as the first-line imaging modality for suspected GCA, its utility in detecting extracranial involvement, such as aortitis, is limited [17]. The pattern of vascular involvement in subclinical GCA appears to be inverse to that observed in classical GCA cohorts [20, 29]. This phenomenon is more obvious in our study and may suggest that the vascular involvement in patients with subclinical GCA is primarily characterized by an isolated extracranial pattern, highlighting the value of PET/CT in detecting this subtype. Although findings of ‘halo’ signs in the axillary and subclavian arteries can aid in identifying certain cases of extracranial subclinical GCA, isolated aortitis may still be overlooked using this method. MRA offers detailed anatomical imaging, but it lacks the ability to distinguish active inflammation from chronic changes. PET-CT, by contrast, provides a comprehensive evaluation of both metabolic activity and structural abnormalities, making it superior for detecting systemic and extracranial vascular involvement. However, PET-CT is less sensitive in detecting inflammation in the temporal arteries and their branches, identifying only approximately one-third of patients with temporal arteritis [30, 31]. Besides, challenges such as radiation exposure and cost remain barriers to widespread adoption. Further prospective studies are needed to refine vascular screening protocols and evaluate the impact of early detection on patient outcomes.

An intriguing finding was that arthralgia appeared to be a protective factor against relapse in patients with PMR. Arthralgia primarily presented as pain and stiffness in the shoulder and pelvic girdles and neck, occasionally with peripheral joint involvement. This clinical presentation aligns with typical periarticular and musculotendinous inflammation in PMR, including such as tendinitis and bursitis as previously demonstrated in imaging studies using US, MRI and PET/CT [2, 32, 33]. Although total Leuven scores were comparable between the groups, FDG uptake in the hips was higher in patients with isolated PMR than in those with subclinical GCA/PMR. This suggests a distinct pattern of inflammation more localized to periarticular structures in the isolated PMR group, which may be associated with a more favourable prognosis. These findings may explain the inverse association between arthralgia and relapse observed in our analysis. However, whether the degree and distribution of joint involvement are independently associated with relapse risk remains unclear and warrants further investigation in larger prospective cohorts.

This study has several limitations. As a retrospective study, it is inherently constrained by potential biases. First, 17 patients were lost to follow-up and excluded, which may have introduced selection bias, particularly if their disease severity differed from those who remained in follow-up. Second, PET/CT findings were available to treating physicians and may have influenced diagnostic and therapeutic decisions. Nonetheless, the comparable baseline glucocorticoid doses between the groups suggest that subclinical GCA was likely under-recognized at the time and that the impact of imaging findings on initial treatment was likely limited. Third, the lack of concurrent US data prevented a direct comparison between the strengths of PET/CT and US for vascular screening. Lastly, although quantitative tools like PETVAS could enhance relapse risk stratification, the limited sample size restricted our ability to conduct multivariable analyses incorporating imaging scores such as PETVAS.

In conclusion, this study highlights the value of ^18^F-FDG PET/CT in identifying subclinical GCA and predicting relapse in PMR patients. PMR patients with subclinical GCA exhibited a high prevalence of extracranial vascular involvement and significantly increased relapse rates, underscoring the importance of vascular screening in PMR management. Additionally, the observation that arthralgia may serve as a protective factor against relapse provides new insights into disease heterogeneity within the PMR spectrum. Future research is warranted to refine imaging protocols, uncover the mechanisms behind arthralgia’s protective effect and develop targeted treatments to improve PMR outcomes.

## Supplementary Material

keaf557_Supplementary_Data

## Data Availability

Further information and requests for clinical data should be directed to and will be fulfilled by the lead contact, Huihui Chi (chh_210@126.com).
