# Supplementary material for: Vascular uptake on 18F-FDG PET/CT predicts relapse in new-onset PMR
Source: Rheumatology (Oxford). 2025 Oct 23;65(1):keaf557. doi: 10.1093/rheumatology/keaf557 (PMC12862371; doi:10.1093/rheumatology/keaf557)
Supplement: keaf557_Supplementary_Data [file keaf557_supplementary_data.docx]

Supplementary materials


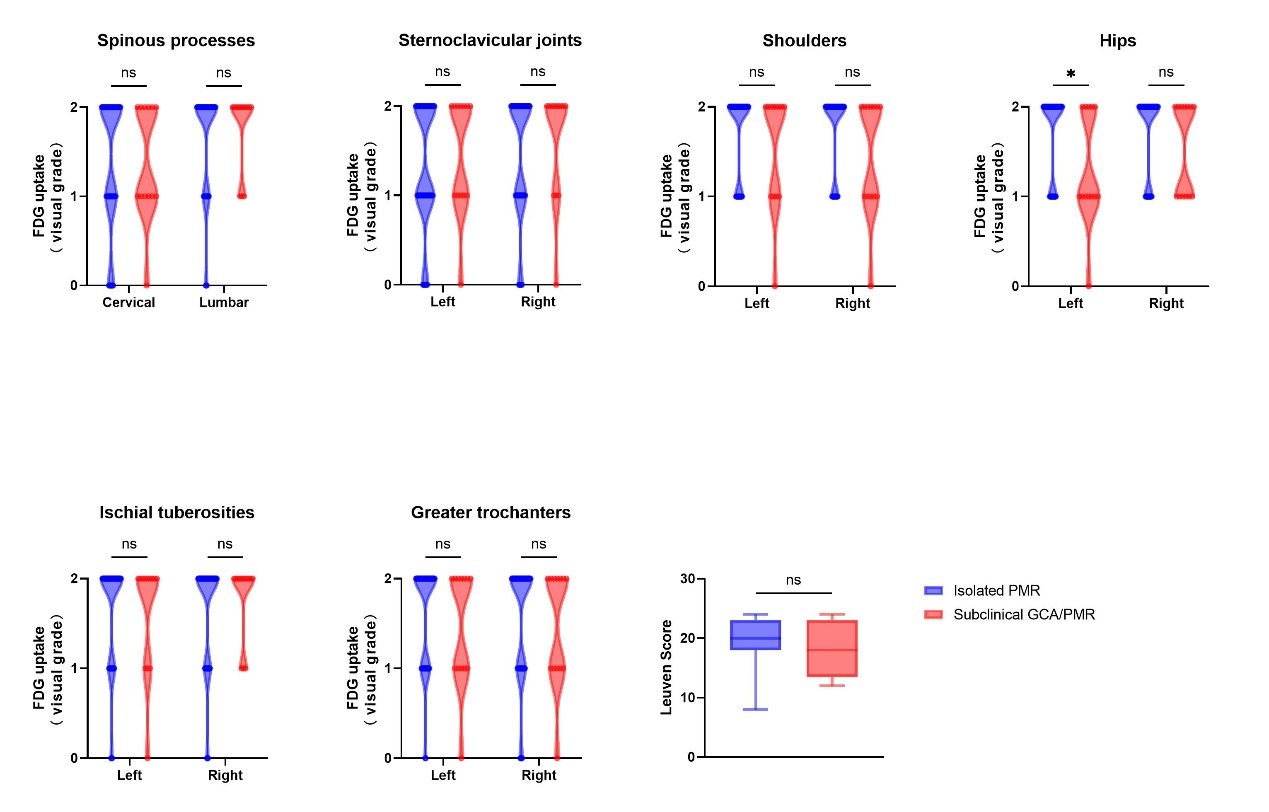


Supplementary Figure S1. Violin plots indicating FDG uptake (visual grade) at 12 musculoskeletal regions and a box plot showing the Leuven scores in patients with isolated PMR (n = 66) and subclinical GCA/PMR (n = 14). Visual grades and Leuven scores of two groups were compared by the Mann-Whitney U test. * p< 0.05.

Supplementary Table S1. The maximum FDG uptake grade across vascular segments for each patient assessed by nuclear medicine physicians.

|  | Physician 1 | Physician 2 | Physician 3 |
| --- | --- | --- | --- |
| P1 | 1 | 1 |  |
| P2 | 1 | 1 |  |
| P3 | 3 | 3 |  |
| P4 | 1 | 1 |  |
| P5 | 0 | 0 |  |
| P6 | 1 | 1 |  |
| P7 | 1 | 1 |  |
| P8 | 1 | 1 |  |
| P9 | 1 | 1 |  |
| P10 | 1 | 1 |  |
| P11 | 1 | 1 |  |
| P12 | 0 | 0 |  |
| P13 | 3 | 3 |  |
| P14 | 1 | 1 |  |
| P15 | 0 | 0 |  |
| P16 | 1 | 1 |  |
| P17 | 0 | 0 |  |
| P18 | 1 | 1 |  |
| P19 | 1 | 1 |  |
| P20 | 1 | 0 |  |
| P21 | 0 | 0 |  |
| P22 | 3 | 3 |  |
| P23 | 0 | 0 |  |
| P24 | 1 | 1 |  |
| P25 | 1 | 1 |  |
| P26 | 0 | 0 |  |
| P27 | 0 | 0 |  |
| P28 | 1 | 1 |  |
| P29 | 0 | 0 |  |
| P30 | 0 | 1 |  |
| P31 | 0 | 0 |  |
| P32 | 1 | 1 |  |
| P33 | 3 | 3 |  |
| P34 | 1 | 1 |  |
| P35 | 1 | 1 |  |
| P36 | 1 | 1 |  |
| P37 | 1 | 1 |  |
| P38 | 1 | 1 |  |
| P39 | 1 | 2 | 1 |
| P40 | 3 | 3 |  |
| P41 | 1 | 1 |  |
| P42 | 3 | 3 |  |
| P43 | 0 | 0 |  |
| P44 | 3 | 3 |  |
| P45 | 1 | 1 |  |
| P46 | 1 | 1 |  |
| P47 | 1 | 1 |  |
| P48 | 0 | 0 |  |
| P49 | 0 | 1 |  |
| P50 | 1 | 1 |  |
| P51 | 1 | 2 | 2 |
| P52 | 0 | 0 |  |
| P53 | 1 | 1 |  |
| P54 | 0 | 0 |  |
| P55 | 0 | 0 |  |
| P56 | 3 | 3 |  |
| P57 | 1 | 1 |  |
| P58 | 1 | 1 |  |
| P59 | 1 | 1 |  |
| P60 | 1 | 1 |  |
| P61 | 1 | 0 |  |
| P62 | 0 | 0 |  |
| P63 | 1 | 1 |  |
| P64 | 3 | 3 |  |
| P65 | 0 | 0 |  |
| P66 | 1 | 1 |  |
| P67 | 1 | 1 |  |
| P68 | 1 | 1 |  |
| P69 | 1 | 2 | 2 |
| P70 | 0 | 0 |  |
| P71 | 2 | 2 |  |
| P72 | 0 | 0 |  |
| P73 | 1 | 1 |  |
| P74 | 1 | 1 |  |
| P75 | 0 | 0 |  |
| P76 | 1 | 1 |  |
| P77 | 1 | 1 |  |
| P78 | 2 | 2 |  |
| P79 | 0 | 0 |  |
| P80 | 3 | 3 |  |
